# Clinical and Biochemical Factors Associated with Delayed Renal Response in Proliferative Lupus Nephritis: A 20-Year Single-Center Multiethnic Cohort Study

**DOI:** 10.3390/biomedicines14030512

**Published:** 2026-02-26

**Authors:** Rozita Mohd, Noor Syazwani Izyan Abdul Rahaman, Lydia Kamaruzaman, Wan Rohaslizan Wan Daud, Muhammad Yusuf Abu Shamsi, Lim Kuan Yee, Ruslinda Mustafar, Abdul Halim Abdul Gafor, Syahrul Sazliyana Shaharir

**Affiliations:** 1 Department of Medicine, Faculty of Medicine, Universiti Kebangsaan Malaysia, Cheras, Kuala Lumpur 56000, Malaysia; rozi8286@gmail.com (R.M.); syazzaizyan@gmail.com (N.S.I.A.R.); lydia.kamaruzaman@ukm.edu.my (L.K.); limkuanyee@gmail.com (L.K.Y.); ruslinda.mustafar@hctm.ukm.edu.my (R.M.); halimgafor@gmail.com (A.H.A.G.); 2 Department of Medicine, Hospital Canselor Tuanku Muhriz, Universiti Kebangsaan Malaysia, Cheras, Kuala Lumpur 56000, Malaysia; rohaslizan@hctm.ukm.edu.my (W.R.W.D.); mdyusof@hctm.ukm.edu.my (M.Y.A.S.)

**Keywords:** ethnic, lupus, renal, remission

## Abstract

**Background/Objectives**: Renal response remains underexplored across ethnic groups in the Asia–Pacific region; Malaysia, being a multiethnic country, provides a unique setting to examine these variations within the same healthcare system. This study was conducted to identify the clinical and biochemical characteristics and factors associated with renal response in Malaysia’s multiethnic LN population. **Methods**: A retrospective cohort study of biopsy-proven proliferative LN episodes between 2000 and 2020 was conducted. Baseline clinical, laboratory, and treatment variables were extracted from medical records. Each relapse was analyzed as a separate episode. Partial and complete renal responses (PRR/CRR) at 6, 12, and 24 months were recorded. Predictors of early complete remission (CR), defined as CRR at 12 months while on prednisolone ≤ 10 mg daily, were identified using a generalized estimating equations (GEE) analysis. **Results**: A total of 212 LN episodes in 145 patients were included. Most episodes occurred in Malay (61.3%), Chinese (34.9%), and Indian (3.8%) patients. The rates of CRR/PRR at 6, 12, and 24 months were 34.9%/35.4%, 23.6%/47.6%, 14.6%/61.3%, respectively. In multivariable GEE analyses, delayed induction (OR 4.19), relapse LN episode (OR 3.91), comorbid hypertension (OR 3.51), and Malay ethnicity (OR 3.23) were associated with delayed complete remission. In contrast, achievement of any remission (partial or complete) at 6 months was protective against delayed CR at 12 months. **Conclusions**: Our findings underscore the importance of early induction therapy and relapse prevention in LN, as both are key predictors of renal response. The observed ethnic disparities warrant confirmation in larger prospective studies to elucidate potential factors and underlying genetic influences of renal response among the diverse ethnic groups.

## 1. Introduction

Systemic lupus erythematosus (SLE) is a chronic, multisystem complex autoimmune disease with the presence of uncontrolled autoreactive antibodies that cause inflammation of tissues and organs. Manifestations of SLE are phenotypically and clinically heterogeneous, and Asian patients tend to have more severe disease with renal involvement. The reported prevalence of SLE in Malaysia is around 40–60/100,000 population [1]. It causes significant impairment in the functional quality of life among young adults [2,3]. Renal involvement among the Malaysian SLE population is high, affecting 40–60% [4,5,6]. Lupus nephritis (LN) is associated with significant morbidity in our cohort, as organ damage accrual was reported in almost half of them [7].

Although mortality in lupus nephritis (LN) has substantially decreased over recent decades [8], the cumulative incidence of developing end-stage renal disease (ESRD) remains significant, ranging from 3% to 11%, 6% to 19%, and 19% to 25% at 5, 10, and 15 years, respectively [9]. In addition, the risk is demonstrated to be higher in developing countries compared with developed countries [9]. Several multiethnic studies have demonstrated that the risk of progression and prognosis of LN may differ between ethnic groups. It is well established that African American and Hispanic populations from the United States are at higher risk of developing severe LN and ESRD [10]. A more severe prognosis was also demonstrated in a Latin-American cohort, as 20% of them were reported to progress to ESRD with a median time of 4.2 (2.0–55.2) months after biopsy [11]. Meanwhile, data from the Australia and New Zealand Dialysis and Transplant Registry (ANZDATA) revealed that men, Māori, and Pacific patients with SLE were more likely to develop ESRD [12]. Consistent with these observations, we previously demonstrated a substantial burden of LN in our multiethnic cohort, reflected by high rates of damage accrual [7].

Despite growing consensus that remission is a desirable treatment target in lupus nephritis (LN), evidence specifically addressing the impact of delayed achievement of remission on renal damage remains limited. Most existing studies emphasize the importance of achieving early remission, whereas the long-term consequences of delayed remission are insufficiently characterized [13,14]. Moreover, existing studies have also largely focused on the presence, prevalence, or durability of remission, demonstrating that sustained remission or prolonged time spent in low disease activity is associated with reduced damage accrual and improved quality of life [15,16,17,18,19]. However, few studies have examined time to first remission as a primary outcome or evaluated whether delayed remission independently increases the risk of subsequent renal damage. In LN specifically, studies conducted in predominantly Caucasian cohorts have shown that a longer time to remission is associated with increased renal damage and poorer long-term renal outcomes [13,20].

The majority of the current published literature examines the associations between renal characteristics and response with the long-term prognosis of LN [21,22,23]. Early complete remission in LN is considered an important protective factor against the development of chronic kidney disease (CKD) and ESRD, [24,25,26] and it was associated with sustained remission at 3 years, reduced organ damage, and lower flare rates [27,28,29]. Our study attempted to compare the baseline clinical characteristics between patients who are likely to achieve early response and delayed response. Malaysia is a multiethnic country that consists predominantly of the Malay ethnic group, followed by Chinese and Indian, so this study also aimed to determine any differences in the disease course and outcome across different Malaysian ethnic groups. Results from this study could lead to better risk stratification and management strategies for patients with LN.

## 2. Materials and Methods

### 2.1. Study Design and Population

This was a single-center retrospective observational cohort study, which included active LN episodes that occurred from 2000 until 2020 in Hospital Canselor Tuanku Muhriz, Universiti Kebangsaan Malaysia (UKM). All LN were confirmed by renal biopsy to have proliferative (Class III or IV) with or without membranous (Class V) LN, according to the International Society of Nephrology/Renal Pathology Society (ISN/RPS) 2003 [30]. Eligible SLE with LN patients were identified from the institution’s SLE database. Convenience sampling was applied, as only cases with medical records retrievable from the Department of Medical Records were included in the study. This study was conducted after obtaining the ethics approval from the Medical Ethics Review Committee (MREC), Universiti Kebangsaan Malaysia (UKM) in June 2020 (FF-2020-281).

Relapse LN episodes, which required another course of induction treatment, were considered as separate episodes. All episodes of active LN must complete at least a 24-month period of observation. LN episodes with incomplete observation and inconsistent follow-up documentation within 24 months after induction treatment were excluded. In addition, LN episodes with documented non-compliance or defaulted follow-up were excluded from the analysis.

### 2.2. Data Collection

Demographic, clinical, laboratory, and biopsy data were retrieved from medical records. Data collected from the medical records included sociodemographic features (gender, ethnicity, age at diagnosis of SLE), LN disease features (renal biopsy classification, first or relapse LN episode), and the presence of comorbidities such as antiphospholipid syndrome, hypertension, diabetes, and dyslipidemia. The baseline characteristics and laboratory values (at the initial proliferative LN active episode prior to induction treatment (T_0_)) that were collected included age, serum creatinine, proteinuria level (measured using urine protein: creatinine index, UPCI), anti-dsDNA (positive/negative), and complement C3 and C4 levels. Proteinuria at 3 and 6 months was also recorded.

Information on the medications or drugs used to treat the active LN episode was recorded. In our institution, the first-line induction protocol for proliferative LN consisted of intravenous pulse methylprednisolone followed by oral prednisolone (0.5 mg/kg/day) with IV cyclophosphamide for 6 months or mycophenolate mofetil (MMF). The second-line induction for refractory or intolerant cases includes calcineurin inhibitor (CNI) or combination MMF and CNI (multi-target) therapy. The immunosuppressive maintenance therapy consisted of either MMF, Ciclosporin A, or azathioprine for 12–18 months. The oral prednisolone was tapered by 5 mg monthly until maintenance at 10 mg daily. Adjunctive therapies, which included intravenous immunoglobulin (IVIG) and plasmapheresis, were also recorded. Delayed induction was defined as initiation of induction therapy for more than 3 months after a clinical diagnosis of active lupus nephritis. Lupus nephritis episodes were stratified into those occurring from 2000–2010 and 2011–2020 to account for temporal changes in lupus nephritis management, particularly following the publication of the Aspreva Lupus Management Study (ALMS) trial in 2009.

### 2.3. Definition of Complete Remission, Relapse, and Outcomes

Definitions for remission and relapse were modified based on the EULAR/ERA-EDTA recommendations for LN 2012 [31] and the 2012 Kidney Disease: Improving Global Outcomes (KDIGO) Clinical Practice Guideline for Glomerulonephritis recommendations [32]. CRR was defined as a urine protein/creatinine ratio (UPCR) of <50 mg/mmol and normal or near-normal (within 10% of normal glomerular filtration rate [GFR] if previously abnormal) renal function. PRR was defined as a ≥50% reduction in proteinuria and normal or near-normal GFR.

In our study, to minimize potential bias from variable background prednisolone doses, we included an oral prednisolone dose of ≤10 mg once daily as a mandatory criterion for complete renal response (CRR) at 12 months. Although the Definitions of Remission in SLE (DORIS) recommend ≤5 mg of prednisolone daily [33] and the Lupus Low Disease Activity State (LLDAS) defines it as ≤7.5 mg daily [34], these guidelines were published after the observation period of our lupus nephritis cohort. Moreover, these recommendations were primarily validated in general SLE populations rather than specifically in LN. Accordingly, prior to these recommendations, our institution’s protocol maintained an oral prednisolone dose of 10 mg daily for at least 3–6 months after achieving complete renal response before initiating tapering. Therefore, our study defined early complete remission (CR) as the ability to achieve CRR at 12 months with oral prednisolone of ≤10 mg daily.

Outcomes of the active LN episodes that were recorded included the presence of CR at 24 months, serum creatinine at 24 months, and the presence of renal damage based on the Systemic Lupus International Collaborative Clinics/American College of Rheumatology (SLICC/ACR) Damage Index [35]. The time to relapse was calculated from the date of remission. Data was censored at loss to follow up, development of end-stage renal disease (ESRD), death, or until 20 November 2023.

### 2.4. Statistical Analysis

Categorical variables were presented using frequencies. For continuous data, the mean (SD) or median with interquartile range (as appropriate) was applied. To investigate the differences in baseline characteristics and renal outcomes between patients with early vs. delayed CR, the *t*-test and Mann–Whitney U test for independent samples (for continuous variables) and the χ^2^ and Fisher’s exact test (for categorical variables) were applied. To account for within-subject correlation due to repeated lupus nephritis episodes within the same individual, generalized estimating equations (GEE) with a binary outcome were used to estimate population-averaged associations. A logit link function was applied and an exchangeable working correlation structure was specified, assuming constant correlation between repeated observations within individuals. Robust (sandwich) variance estimators were used to obtain valid standard errors. All variables with *p*-values < 0.1 in univariable analysis and LN episodes from 2000–2010 were additionally included in the model to account for potential confounding. Missing laboratory data, such as antiphospholipid antibodies, anti-dsDNA, and complement C3/C4 levels pre-induction treatment, were handled using complete-case analysis.

Statistical analyses were performed using SPSS version 27.

## 3. Results

### 3.1. Baseline LN Patient Characteristics

A total of 185 proliferative LN patients were identified from the institution’s database. Of these, 40 patients were excluded. A total of 32 patients were excluded due to incomplete medical records: 3 because of insufficient observation periods, 2 who defaulted on follow-up, and 2 who underwent renal biopsy and initiated treatment at another institution. Consequently, 145 patients were included in the final analysis. The majority of the cohort comprised patients of the Malay ethnic group, followed by Chinese and Indian, reflecting the ethnic composition in Peninsular Malaysia. More than half of them had concomitant hematological manifestations, followed by musculoskeletal and mucocutaneous lupus. No significant differences in clinical characteristics were observed among the various ethnic groups in this cohort, as illustrated in Table 1.

### 3.2. Lupus Nephritis Characteristics and Renal Response at 6, 12, and 24 Months in Different Ethnic Groups

A total of 212 active lupus nephritis episodes were identified among the 145 patients. At baseline, LN episodes in Malay patients were characterized by a higher prevalence of nephrotic-range proteinuria and antiphospholipid syndrome (APLS) compared to non-Malay patients. At 6 months, complete renal response (CRR) and partial renal response (PRR) were achieved in 35.4% and 34.9% of all LN episodes, respectively. The overall remission rate (CRR or PRR) at 6 months was significantly lower among Malay patients compared to non-Malay patients. At 12 months, Malay patients continued to demonstrate significantly lower rates of both CRR and PRR compared to Chinese and Indian patients. By 24 months, LN episodes in the Malay group had a significantly lower rate of complete renal response compared to the non-Malay group (Table 2).

### 3.3. Factors Associated with Early Renal Response

At 12 months, a total of 47.6% of patients achieved early CR (n = 101), and any renal response (CR or PR) at 6 months predicted this early CR response. Malay patients had a lower rate of early CR compared to the Chinese and Indian patients. Early CR was also significantly lower in LN relapse episodes in patients with antiphospholipid syndrome (APLS), baseline nephrotic-range proteinuria, baseline positive anti-dsDNA, and hypertension at baseline (T0) and as a comorbidity. The longer interval of induction treatment initiation from the time of LN episode diagnosis was associated with delayed CR.

The CR rate at 24 months was 60.4% (n = 128), and early CR (at 12 months) predicted this outcome (Table 3). LN episodes with delayed CR had significantly higher creatinine at 24 months, CKD at 24 months, and a shorter interval to relapse. Early ESRD at 24 months occurred in all 3 LN episodes with delayed CR.

A generalized estimating equation (GEE) model was used to identify the factors associated with delayed CR (inability to achieve CR at 12 months) in LN: Malay ethnicity, comorbid hypertension, LN relapse episodes, and longer interval of induction treatment from LN diagnosis. Any remission at 6 months protected against delayed CR, as illustrated in Table 4.

## 4. Discussion

The management of patients with SLE has improved over recent decades; unfortunately, LN remains a poor prognostic factor, as it is associated with significant morbidity as well as mortality due to ESRD, infection, and cardiovascular disease [36,37]. The rate of CKD and/or ESRD is still substantially significant, affecting 10–30% of patients [23]. Responses to immunosuppressive therapy and disease course vary across ethnic groups, as demonstrated in the ALMS [38] and other multiethnic cohort studies [39]. Malaysia has the advantages of a diverse population comprising major Asian ethnic groups (i.e., Malay, Chinese, and Indian), providing an opportunity to study outcomes across these ethnic groups within the same environment and healthcare system.

Approximately two-thirds of LN episodes in our cohort achieved either a complete (35.4%) or a partial (34.9%) renal response at 6 months. The reported remission rates at 6 months in other Asia–Pacific cohorts varied widely, ranging from 20% to 80%, reflecting differences across regions and ethnic groups [21,40,41]. The highest renal response was observed in the Singaporean LN cohort, with a complete remission rate of 81.4% at 6 months. However, the Singaporean LN cohort primarily comprised Chinese patients, and this finding supports earlier studies that reported favorable renal responses with more than 80% CR rates among Chinese LN patients [42,43]. In contrast, other Asian LN cohorts reported lower 6-month CR rates, i.e., 45% at 6 months in the Egyptian cohort [44], 39% in the Saudi Arabian cohort [45], and 28.3% among Siamese LN patients who received MMF [40].

Our study found that less than half of LN patients (46.7%) achieved the complete remission target at 12 months. This was similar to the multiethnic Australian LN cohort, which comprised 52% Asian and 32% Caucasian patients and reported a 12-month CR rate of 47% [46]. In contrast, the Indian LN cohort and Mainland Chinese cohort reported higher CR rates of 63.75% [47] and 60.7% [48], respectively. However, these studies did not take into consideration the background oral prednisolone use; hence, this may explain the lower CR rate in our cohort, as we included the mandatory definition of oral prednisolone ≤ 10 mg daily to reflect the maintenance dose of background oral corticosteroids in LN. A Hispanic LN cohort from Mexico demonstrated considerably lower complete renal response (CRR) rates of 22.3%, 40.5%, and 51.6% at 6, 12, and 24 months, respectively [49]. Similar to the Hispanic cohort, LN patients in Ethiopia reported a low CR rate of 35.1% within 12 months of induction treatment. In contrast, a European cohort reported higher CR rates of 50.0% and 68.3% at 6 and 12 months, respectively [50]. Despite lower early CR rates in our cohort, the long-term CR at 24 months was 60.4%, slightly higher than reported in the Hopkins Lupus cohort, where CR at 24 months was approximately 40% and 59% based on the modified Belimumab International Lupus Nephritis Study (mBLISS-LN) and modified Aspreva Lupus Management Study (mALMS) criteria, respectively [23].

Our study is the first to report that lupus nephritis (LN) episodes among Malay patients are associated with poorer responses to standard LN therapy, as reflected by higher rates of failure to achieve complete remission at 12 months. Ethnic background has been shown to significantly influence disease severity and treatment outcomes in systemic lupus erythematosus (SLE). Post hoc analyses of major randomized controlled trials have demonstrated differential treatment responses across ethnic groups, indicating that ethnicity-related disparities persist even within rigorously controlled trial settings. In particular, African American and Hispanic patients have shown reduced response to intravenous cyclophosphamide but more favorable outcomes with mycophenolate mofetil, especially in LN [51]. Subgroup analysis of the LUNAR trial further suggested ethnic variation in treatment response, with Black patients demonstrating higher partial renal response rates with rituximab [52]. While genetic susceptibility may partly account for these differences, ethnicity may also act as a surrogate for unmeasured factors such as socioeconomic status, education level, health literacy, medication adherence, and access to care. These factors were not evaluated in our study and thus warrant further investigation to better elucidate the mechanisms underlying ethnic disparities in renal treatment response.

Our study has also identified several other factors associated with delayed remission, including comorbid hypertension and relapse LN episodes. A multicenter Italian LN study demonstrated that the absence of arterial hypertension was one of the independent predictors of remission [53]. In addition, the presence of hypertension has been demonstrated to be associated with worse renal outcomes in LN, such as the doubling of serum creatinine at 5–10 years [14] and chronic kidney disease [54]. Our study findings also highlighted that relapsed LN episodes predict poor response to treatment and delayed CR, and this finding concurs with other studies [41,55]. Previous studies have also demonstrated that renal flares may be an independent predictor of incident and progressive CKD and ESRD in LN [21,47,56,57]. Therefore, apart from early remission, maintenance of this response is crucial to prevent adverse outcomes in LN. Our study also showed that the ability to achieve any renal response (CRR or PRR) at 6 months is important, as it predicts CR at 12 months. This was also consistent with the Hispanic LN cohort study, which showed that renal response at 6 months predicted the CRR outcome [58].

Our study further underscores the pivotal importance of early diagnosis and timely initiation of treatment, as delays in renal biopsy and longer intervals between renal biopsy and induction therapy were both associated with poorer renal outcomes [59,60,61]. Current practice often reserves renal biopsy for patients with isolated proteinuria exceeding 1 g/day or urine protein–creatinine ratio (UPCR) > 1. However, this threshold may warrant reconsideration, as data from the Accelerating Medicines Partnership (AMP) lupus nephritis study demonstrated that 85.2% of patients with UPCR < 1 had histological evidence of proliferative and/or membranous lupus nephritis [62]. Recent evidence suggests that non-White race and lower socioeconomic status (food insecurity and unstable housing) are associated with delays in undergoing renal biopsy. As our study was retrospective, we were unable to capture the underlying causes of delayed induction therapy, as these factors were not consistently documented. This highlights an important area for future research to better understand and address barriers to timely diagnosis and treatment.

The use of hydroxychloroquine (HCQ) was demonstrated to be associated with early renal response in the Hispanic LN cohort and in many other studies [49,63,64]. However, in our study, the protective effect of hydroxychloroquine against delayed remission did not reach statistical significance in the GEE model. This finding may be attributable to the limited sample size and the inability to reliably assess medication adherence in this retrospective analysis. There are several other limitations in our present study; although we excluded LN episodes with documented non-compliance, treatment adherence could not be confirmed. In addition, several confounding variables that were not recorded may influence the outcomes, such as the socioeconomic and educational background of the patients.

Nevertheless, our study is the first to highlight ethnic disparities in renal responses among different Asian ethnic groups, which warrants further investigations to identify genetic and non-genetic factors [65]. Our findings suggest that renal response and outcomes may not be uniform across Asian populations, highlighting the need for greater ethnic diversity within Asian cohorts in clinical trials. The study also underscores the importance of early and timely induction therapy following the diagnosis or strong clinical suspicion of lupus nephritis. A treat-to-target approach aimed at achieving at least partial remission by 6 months and effective maintenance therapy are important strategies to prevent relapse, which predicts poor renal response to the standard LN therapies. Future studies should further explore socioeconomic, cultural, and genetic factors that may influence treatment response in lupus nephritis.

## 5. Conclusions

In conclusion, although Malays are the major ethnic group in Malaysia, the renal response among them was lower compared to Chinese and Indians. Other factors associated with poor renal response include relapsed LN episodes, hypertension, and delayed induction treatment.

## Figures and Tables

**Table 1:** Baseline characteristics of the multiethnic lupus nephritis patients (LN) included in the study (*n* = 145).

Variable	Mean ± SD, n (%)				
Ethnic	All *n* = 145 (100%)	Malay *n* = 93 (64.1%)	Chinese *n* = 45 (31.0%)	Indian *n* = 7 (4.8%)	*p*-Value
Age of SLE diagnosis, (years)	24.8 ± 8.0	24.9 ± 7.9	25.2 ± 9.1	23.6 ± 6.1	0.80
SLE duration (months)	17.3 ± 6.1	16.6 ± 5.9	19.0 ± 6.2	16.0 ± 2.2	0.07
Median (IQR) follow-up duration (months)	160.5 (24–263)	149.5 (24–263)	179 (24–263)	130 (38–182)	0.23
Gender					
Female	126 (86.9)	80 (87.0)	41 (89.1)	6 (85.7)	0.88
Male	19 (13.1)	13 (14.0)	5 (10.9)	1 (14.3)	
Other manifestations					
Hematological	109 (75.2	72 (77.4)	33 (71.7)	5 (71.4)	0.74
Musculoskeletal	94 (64.8)	64 (68.8)	25 (54.3)	6 (85.7)	0.12
Mucocutaneous	76 (52.4)	50 (53.8)	22 (47.8)	4 (57.1)	0.78
Neuropsychiatry	27 (18.6)	17 (18.3)	10 (21.7)	0 (0)	0.38
Serositis	22 (15.2)	12 (12.9)	8 (17.4)	2 (28.6)	0.47
Antiphospholipid	15 (10.3)	12 (13.5)	2 (2.2)	1 (16.7)	0.12
Any renal damage	22.8 (33)	28.0 (26)	15.6 (7)	0	0.09
CKD	13.8 (20)	18.3 (17)	6.7 (3)	0	0.10
ESRD	9.0 (13)	9.7 (9)	8.9 (4)	0	0.69

**Table 2:** LN characteristics at baseline (T_0_) and renal response according to ethnicities at 6 months (after induction), 12 months, and 24 months.

LN Characteristics	All *n* = 212	Malay *n* = 130	Chinese *n* = 74	Indian *n* = 8	*p*-Value
Age T_0_, years	29.3 ± 9.4	28.6 ± 8.1	30.6 ± 11.4	27.8 ± 9.4	0.40
Mean interval from LN diagnosis to induction, (months)	3.0 ± 3.9	3.1 ± 3.7	3.1 ± 4.6	1.7 ± 1.8	0.63
LN Class, (n, %)					
Class III ± V	45.8 (97)	46.2 (60)	41.9 (31)	75 (6)	0.20
Class IV ± V	54.3 (115)	53.5 (70)	58.1 (43)	25 92)	
Mean creatinine T_0_, (µmol/L)	95.0 ± 65.9	99.6 ± 73.2	88.9 ± 53.8	76.9 ± 52.6	0.40
Nephrotic proteinuria T_0_ (n, %)	56.1 (119)	61.5 (80)	48.6 (36)	37.5 (3)	0.11
Acute kidney injury T_0_ (n, %)	25.5 (54)	27.7 (36)	20.3 (15)	37.5 (3)	0.37
Hypertension T_0_ (n, %)	53.8 (114)	58.5 (76)	44.6 (33)	62.5 (5)	0.14
APLS	10.4 (22)	15.4 (20)	1.4 (1)	12.5 (1)	0.007
Diabetes mellitus	8.5 (18)	10.8 (14)	4.1 (3)	12.5 (1)	0.23
Hypertension	28.8 (61)	33.1 (43)	20.3 (15)	37.5 (3)	0.13
Dyslipidemia	15.6 (33)	16.9 (22)	13.5 (10)	12.5 (1)	0.79
Renal response at 6 months (n, %)					
No remission ^$^	29.7 (63)	35.4 (46)	21.6 (16)	12.5 (1)	0.102
CRR ^&^	35.4 (75)	30.0 (39)	41.9 (31)	62.5 (5)	0.061
PRR	34.9 (74)	34.6 (45)	36.5 (27)	25.0 (2)	-
Any remission (CRR and PRR) ^£^	70.5 (149)	64.0 (84)	78.4 (58)	87.5 (7)	0.065
Renal response at 12 months (n, %)					
No remission ^$^	28.8 (61)	36.9 (48)	16.2 (12)	12.5 (1)	0.004
CRR ^&^	47.6 (101)	37.7 (49)	63.5 (47)	62.5 (5)	0.001
PRR	23.6 (50)	25.4 (33)	20.3 (15)	25.0 (2)	-
Any remission	71.2 (151)	63.1 (82)	83.8 (62)	87.5 (7)	0.004
Renal response at 24 months (n, %)					
No remission ^$^	24.1 (51)	26.9 (35)	21.6 (16)	0.0 (0)	0.13
CRR ^&^	61.3 (130)	55.4 (72)	68.9 (51)	87.5 (7)	0.049
PRR	14.6 (31)	17.7 (23)	9.5 (7)	12.5 (1)	-
Any remission ^£^	75.9 (161)	73.1 (95)	78.4 (58)	100.0 (8)	0.19
Sustained CR	42.9 (91)	34.6 (45)	55.4 (41)	62.5 (5)	0.008

CRR = Complete renal response, PRR = Partial renal response, CR = Complete remission, T_0_ = Time prior to induction treatment of proliferative LN. ^$^ No remission vs. CRR vs. PRR, ^&^ CRR vs. others (PRR and no remission), ^£^ Any remission (CRR and PRR) vs. no remission.

**Table 3:** Comparisons of characteristics between early and delayed complete remission (CR) in lupus nephritis (LN) episodes.

Variable	Delayed CR *n* = 111 (52.4%)	Early CR *n* = 101 (47.6%)	*p*-Value
LN year			
2000–2010	54.9 (62)	56.6 (56)	0.89
>2010–2020	45.1 (51)	43.4 (43)	
Age T_0_, years	29.5 ± 8.9	29.0 ± 9.8	0.98
Age onset SLE, years	24.7 ± 7.6	24.3 ± 8.1	0.29
Ethnic, % (n)			
Malay	73.0 (81)	48.5 (49)	0.001
Chinese	24.3 (27)	46.5(47)	
Indian	2.7 (3)	5.0 (5)	
Gender, % (n)			
Female	88.5 (98)	90.1 (91)	0.83
Male	11.7 (13)	9.9 (10)	
APLS	15.3 (17)	5.0 (5)	0.01
Comorbidities			
Diabetes mellitus	10.8 (12)	5.9 (6)	0.23
Hypertension	41.4 (46)	14.9 (15)	<0.001
Dyslipidemia	24.3 (27)	5.9 (6)	<0.001
LN Classification			
Class III ± V	47.7 (53)	43.6 (44)	0.58
Class IV ± V	58.6 (58)	50.4 (57)	
Presence of crescents, % (n)	35.7 (30)	31.3 (31)	0.53
Presence of global sclerosis, % (n)	25.0 (21)	44.0 (44)	0.009
Activity index	7 (0–21)	7 (0–19)	0.89
Chronicity index	2 (0–18)	3 (0–16)	0.09
Creatinine T_0_, µmol/L	89.0 ± 59.1	100.1 ± 70.9	0.23
Albumin T_0_, g/L	28.9 ± 8.9	29.8 ± 8.2	0.43
Complement C3 T_0_ (n = 164)	50.5 ± 29.1	57.6 ± 29.5	0.09
Complement C4 T_0_ (n = 164)	10.5 ± 8.3	11.6 ± 8.3	0.37
Anti-dsDNA positive T_0_ (n = 203)	72.9 (78)	85.4 (82)	0.04
Nephrotic proteinuria T_0_	63.1 (70)	48.5 (49)	0.04
Acute kidney injury T_0_	25.2 (28)	25.7 (26)	1.00
Hypertension at baseline T_0_	68.5 (76)	48.5 (49)	0.003
Relapse LN episode	55.9 (62)	39.6 (40)	0.009
Interval from LN diagnosis to induction, months	4.2 ± 4.9	1.7 ± 2.1	<0.001
Induction CYC, % (n)	64.0 (71)	69.3 (70)	0.48
Hydroxychloroquine	57.4 (58)	45.0 (50)	0.08
Maintenance MMF	41.4 (46)	59.4 (60)	0.01
Maintenance CyA	24.3 (27)	27.7 (28)	0.64
Maintenance Azathioprine	0.9 (1)	4.0 (4)	0.19
Any remission (CRR and PRR) at 6 months	48.6 (54)	94.1 (95)	<0.001
CR at 24 months	35.1 (39)	90.1 (91)	<0.001
Creatinine at 24 months	110.1 ± 120.9	69.1 ± 18.7	0.003
CKD at 24 months	9.0 (10)	2.0 (2)	0.04
ESRD at 24 months	2.7 (3)	0 (0)	0.25
Interval to relapse, months	33.2 ± 44.8	80.9 ± 58.8	<0.001

CKD = Chronic kidney disease, CR = complete remission, CyA = Ciclosporine A, CYC = Cyclophosphamide, ESRD = End-stage renal disease, LN = Lupus nephritis, MMF = Mycophenolate mofetil, CRR = Complete renal response, PRR = Partial renal response, SLE = Systemic lupus erythematosus, T_0_ = Time prior to induction treatment of proliferative LN.

**Table 4:** Generalized estimating equation model of factors associated with delayed complete remission.

Factors	B	OR (95% C.I)	*p*-Value
Any remission at 6 months	−2.34	0.10 (0.04–0.26)	<0.001
Comorbid hypertension	1.26	3.51 (1.36–9.05)	0.009
Malay	1.17	3.23 (1.35–7.33)	0.008
Relapse LN episode	1.36	3.91 (1.42–10.78)	0.008
Delayed induction *	1.43	4.19 (1.21–14.48)	0.02
Hydroxychloroquine	−0.56	0.57 (0.25–1.31)	0.19
Nephrotic proteinuria T_0_	0.52	1.68 (0.68–4.15)	0.26
Hypertension T_0_	0.23	1.26 (0.56–2.87)	0.58
Anti-dsDNA positive T_0_	−0.58	0.56 (0.17–1.84)	0.33
Complement C3 T_0_	−0.01	0.99 (0.97–1.01)	0.49
MMF maintenance	−0.75	0.47 (0.19–1.14)	0.09
Antiphospholipid syndrome	−0.21	0.81 (0.19–3.47)	0.99
Dyslipidemia	0.46	1.59 (0.56–4.46)	0.39
Global sclerosis present	0.39	1.48 (0.56–3.94)	0.43

OR = Odd ratio, LN = Lupus nephritis, MMF = Mycophenolate mofetil, T_0_ = Time prior to induction treatment of proliferative LN. * Delayed induction: Induction treatment >3 months after active LN diagnosis.

## Data Availability

The original contributions presented in this study are included in the article. Further inquiries can be directed to the corresponding author.

## Abbreviations

The following abbreviations are used in this manuscript:

APLS	Antiphospholipid Syndrome
CR	Complete remission
CRR	Complete renal response
PRR	Partial renal response
ESRD	End-Stage Renal Disease
GEE	Generalized estimating equations
GFR	Glomerular filtration rate
CNI	Calcineurin inhibitor
IVIG	Intravenous immunoglobulin
MMF	Mycophenolate mofetil
SLE	Systemic lupus erythematosus
SLICC/ACR	The Systemic Lupus International Collaborating Clinics/American College of Rheumatology
UPCR	Urine protein: creatinine ratio
